# Erwachsene mit angeborenen Herzfehlern im Notaufnahmedienst: Was ist zu beachten?

**DOI:** 10.1007/s00063-020-00752-6

**Published:** 2020-10-25

**Authors:** J. Mair, G.-P. Diller, H. Geiger, M. Greutmann, G. Hessling, D. Tobler

**Affiliations:** 1 Universitätsklinik für Innere Medizin III – Kardiologie und Angiologie, Medizinische Universität Innsbruck, Anichstraße 35, 6020 Innsbruck, Österreich; 2 Klinik für Kardiologie III – Angeborene Herzfehler und erworbene Klappenerkrankungen, Universitätsklinikum Münster, 48149 Münster, Deutschland; 3 Abteilung Interne II – Kardiologie, Ordensklinikum Linz GmbH – Barmherzige Schwestern, 4010 Linz, Österreich; 4 Universitäres Herzzentrum, Kardiologie, Universitätsspital Zürich, 8091 Zürich, Schweiz; 5 Abteilung für Elektrophysiologie, Deutsches Herzzentrum München, Klinik an der Technischen Universität München, 80636 München, Deutschland; 6 Kardiologie, Universitätsspital Basel, 4031 Basel, Schweiz

**Keywords:** Angeborene Herzfehler, Erwachsene, Notfall, Herzinsuffizienz, Rhythmusstörung, Adult, Congenital heart disease, Emergency, Heart failure, Arrhythmia

## Abstract

Die Patientengruppe der Erwachsenen mit angeborenen Herzfehlern (EMAH) ist mittlerweile bereits größer als die der Kinder mit angeborenen Herzfehlern. EMAH-Patienten weisen auch nach Reparaturoperationen oft komplexe pathophysiologische und anatomische Verhältnisse auf. Bei Komplikationen kann es sehr rasch zu Notfallsituationen auch bei ansonsten asymptomatischen oder nur wenig symptomatischen Patienten kommen. Gemessen an der Gesamtzahl der Patienten, die von Notärzten und in den Notaufnahmen versorgt werden, sind EMAH-Notfallsituationen nach wie vor sehr selten. Diese Übersicht soll die Notfallbetreuung von EMAH-Patienten erleichtern. Für ca. zwei Drittel aller Notfälle sind Rhythmusstörungen und akute Herzinsuffizienz verantwortlich. Rhythmusstörungen müssen in der Regel zügig terminiert werden, weil sie unbehandelt rasch zur kardialen Dekompensation führen können. Bei Scheitern der medikamentösen Therapie oder hämodynamischer Instabilität müssen EMAH-Patienten mit tachykarden Rhythmusstörungen rasch elektrisch kardiovertiert werden. Symptomatische Bradykardien können eine rasche Schrittmacherversorgung erforderlich machen. Aufgrund der komplexen Anatomie kann das Einschwemmen eines transvenösen Interim-Schrittmachers bei einzelnen Vitien unmöglich sein. Die akute kardiale Dekompensation bei EMAH ist oft durch ein akutes Rechtsherzversagen verursacht. Weitere relativ häufige Aufnahmegründe sind Infektionen, Synkopen, Thromboembolien und Aortendissektion. Der Herzpass der Patienten informiert über das vorliegende Vitium. Die umgehende Kontaktaufnahme mit dem behandelnden EMAH-Zentrum wird dringend empfohlen.

## Abstract (en)

The number of adults with congenital heart disease (ACHD) already exceeds the number of children with congenital heart disease in the industrialized world. ACHD patients often show complex pathophysiology and anatomy even after reparative cardiac surgery. In case of complications patients may rapidly deteriorate and become unstable, even when they were asymptomatic or had only mild symptoms before the onset of the complication. Compared to all patients seen by emergency physicians, emergencies in ACHD patients are still rare. This review is aimed to guide management in ACHD emergency situations. Approximately two-thirds of all emergency admissions are caused by arrhythmias or acute heart failure. Sustained arrhythmias may rapidly lead to acute cardiac decompensation in ACHD patients. If medical treatment fails or patients present in hemodynamically unstable conditions, prompt electrical cardioversion is mandatory. Symptomatic bradycardia may require urgent pacemaker implantation. Depending on the underlying heart defect, placement of temporary transvenous pacemaker leads may be impossible. Acute heart failure in ACHD patients is often caused by acute right heart failure. Other more frequent emergencies are infections, syncope, thromboembolic events, and aortic dissection. It is highly recommended to contact the tertiary care center that follows the patient regularly early in case of patient presentation to the emergency room.

## Infobox Mitarbeit der Autoren in einschlägigen Arbeitsgruppen der jeweiligen nationalen kardiologischen Gesellschaften

- J. Mair ist Mitglied des Nukleus der Arbeitsgruppe „Angeborene und erworbene Herzfehler“ der Österreichischen Kardiologischen Gesellschaft
- G.-P. Diller ist Sprecher der Arbeitsgruppe „Kongenitale Herzfehler im Erwachsenenalter“ der Deutschen Gesellschaft für Kardiologie
- H. Geiger ist Leiter der Arbeitsgruppe „Angeborene und erworbene Herzfehler“ der Österreichischen Kardiologischen Gesellschaft
- M. Greutmann ist Past-President der Arbeitsgruppe für „Erwachsene und Teenager mit angeborenen Herzfehlern“ der Schweizerischen Gesellschaft für Kardiologie
- D. Tobler ist Mitglied der Arbeitsgruppe für „Erwachsene und Teenager mit angeborenen Herzfehlern“ der Schweizerischen Gesellschaft für Kardiologie

Die großen Fortschritte insbesondere in der Kinderherzchirurgie und postoperativen Intensivmedizin der letzten Jahrzehnte haben zu einer eindrucksvollen Verbesserung der Lebenserwartung von Kindern auch mit komplexen angeborenen Herzfehlern geführt, sodass die große Mehrheit der Patienten (≥85 %) heute das Erwachsenenalter erlebt. In den westlichen Industriestaaten ist mittlerweile die Patientengruppe der Erwachsenen mit angeborenen Herzfehlern (EMAH) größer als die der Kinder mit angeborenen Herzfehlern. Die geschätzte Zahl der mittel- oder hochkomplexen EMAH-Patienten, die in den deutschsprachigen Ländern leben, liegt bei ca. 275.000 [1]. Ihre Zahl wird in den nächsten Jahrzehnten noch weiter ansteigen. EMAH-Patienten mit komplexen Vitien weisen auch nach Reparaturoperationen oft komplexe pathophysiologische und anatomische Verhältnisse auf. Bei Auftreten von Komplikationen kann es bei Betroffenen rasch zu Notfallsituationen mit hämodynamischer Instabilität kommen, auch wenn die Patienten zuvor asymptomatisch oder kaum symptomatisch waren. Somit entsteht eine immer größer werdende Patientengruppe mit reparierten oder palliativ behandelten Herzfehlern mit spezifischen Problemen, die sich mit speziellen Notfallsituationen den Notärzten, Notfall- und Intensivmedizinern ohne spezifische EMAH-Kenntnisse oft mit unerwartet komplexen Problemen präsentieren. Laut einer jüngeren Umfrage fühlen sich jedoch viele Notfallmediziner aufgrund mangelhafter Ausbildung in diesem Spezialgebiet in der Akutbehandlung dieser seltenen Notfälle überfordert [2]. Daher ist es wichtig, dass alle EMAH-Patienten einen Herzpass besitzen und mitführen (in Papierform oder auch elektronisch gespeichert am Mobiltelefon), in dem die Diagnosen und Voroperationen und die Telefonnummern zur Kontaktaufnahme mit dem betreuenden EMAH-Zentrum angegeben sind. Mit dieser Übersicht wollen wir die Betreuung der EMAH-Patienten mit akuten Notfällen erleichtern, wobei wir aus Platzgründen nur die wichtigsten Notfallsituationen abhandeln können und auf die umfangreichen Richtlinien zur Nachsorge der einzelnen spezifischen EMAH-Krankheitsbilder der Europäischen Kardiologischen Gesellschaft (ESC) und die rezenten Deutschland-Österreich-Schweiz(DACH)-Behandlungsrichtlinien verweisen [1, 3]. Eine von Kaemmerer et al. publizierte Studie [4] gibt eine gute Übersicht über die Häufigkeit der verschiedenen, zu erwartenden Notfälle, die zu einer Krankenhausaufnahme an ein EMAH-Zentrum führten. Die häufigsten Aufnahmegründe waren Rhythmusstörungen (37 %), Herzinsuffizienz (26 %), Infektionen inklusive Endokarditis (11 %), Synkopen, Thromboembolien und Aortendissektion. Somit waren Rhythmusstörungen und Herzinsuffizienz für ca. zwei Drittel aller Notfälle verantwortlich. Es ist zu beachten, dass Rhythmusstörungen eine wichtige Ursache der akuten Herzinsuffizienz bei EMAH-Patienten sind. Etwa 70 % aller Notfälle traten bei bisher asymptomatischen oder nur wenig symptomatischen Patienten auf [4].

## Klassifizierung der EMAH-Patienten, häufige Vitien und Voroperationen

**Figure 1:**
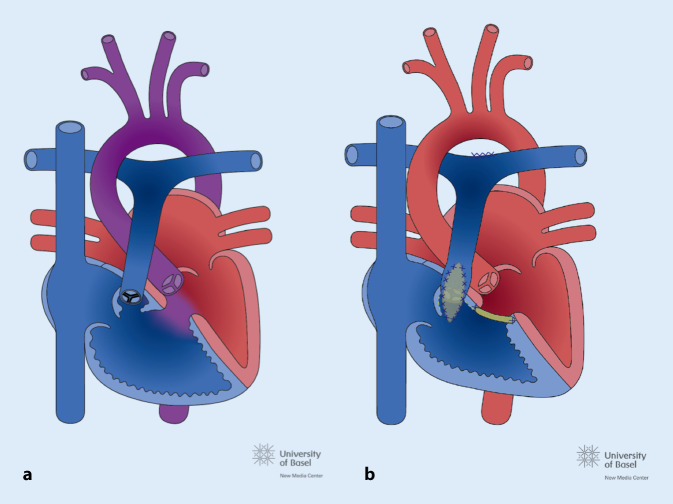

Angeborenen Herzfehler werden üblicherweise in 3 Komplexitätsstufen eingeteilt (Tab. 1): einfache, mittelkomplexe und komplexe Vitien. Ein Grundwissen zur normalen Physiologie und kardiopulmonalen Anatomie des einzelnen EMAH-Patienten ist auch für die Akutversorgung wichtig, insbesondere z. B. bei einem univentrikulären Kreislauf (Abb. 1) oder bei einer höhergradigen pulmonalen Hypertonie. Häufige mittelkomplexe und komplexe Vitien (Abb. 1, 2 und 3) und durchgeführte, häufige Voroperationen sind in den Tab. 2 und 3 aufgelistet.

**Figure 2:**
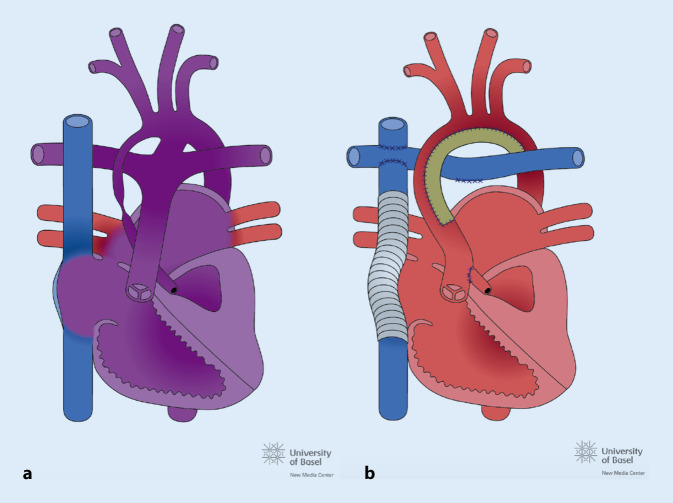

**Table 1:** 

Komplexität des Vitiums	Natives Vitium	Operiertes/interveniertes Vitium
*Einfach*	Mittelgradige kongenitale isolierte Klappenvitien	Z. n. Ductus-arteriosus-Botalli-Verschluss
	Isolierter ASD II	Korrigierter ASD mit oder ohne kleinem Restdefekt
	Kleiner VSD	Korrigierter VSD mit oder ohne kleinem Restdefekt
*Mittelkomplex*	ASD I, Sinus-venosus-ASD	Z. n. Op. bei Fallot-Tetralogie
	Hochgradige kongenitale Klappenvitien	Z. n. Korrektur einer Aortenisthmusstenose
	Offener Ductus arteriosus Botalli	Z. n. Op. bei Transposition der großen Arterien
	Ausflusstraktobstruktionen im rechten und linken Ventrikel	–
*Komplex*	Zyanotische Vitien	Conduits (mit oder ohne Klappe)
	Eisenmenger-Syndrom	Z. n. Fontan mit univentrikulärem Kreislauf

*ASD* Vorhofseptumdefekt, *VSD* Ventrikelseptumdefekt, *Z.* *n.* Zustand nach, *Op.* Operation

**Figure 3:**
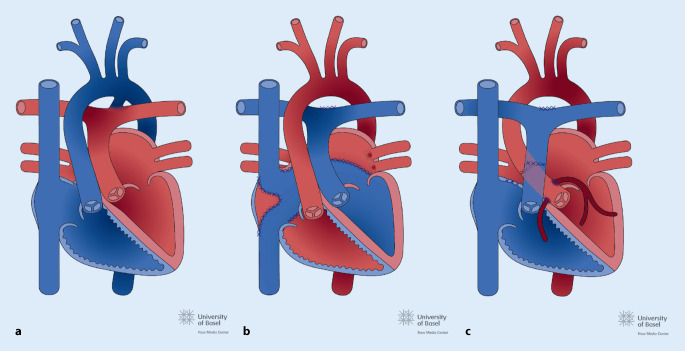

**Table 2:** 

Vitium	Häufigkeit*	Pathophysiologie
*Fallot-Tetralogie *(TOF): Abb. 2	6	Subpulmonalstenose, Hypoplasie der PK und Pulmonalstrombahn (unterschiedlich ausgeprägt), VSD, nach rechts verlagerte, über dem VSD reitende Aorta, RV-Hypertrophie Zyanose
*Transposition der großen Arterien* (TGA): Abb. 3	5	Die Aorta ist mit dem RV und die Pulmonalarterie mit dem LV verbunden, für das Überleben ist ein ASD, VSD oder persistierender PDA notwendig Zyanose Notfallintervention: Rashkind-Ballon-Atrioseptostomie zur Sicherung des Überlebens nach der Geburt
*Univentrikuläres Herz:* Abb. 1	1–3	Anatomischer oder funktionell singulärer Ventrikel z. B. bei Mitral- oder Trikuspidalklappenatresie, hypoplastisches Linksherzsyndrom

*VSD* Ventrikelseptumdefekt, *RV* rechter Ventrikel, *LV* linker Ventrikel, *ASD* Vorhofseptumdefekt, *PDA* persistierender Ductus arteriosus Botalli, *PK* Pulmonalklappe

*In % aller kongenitalen Vitien

**Table 3:** 

Op.-Name	Ziel	Durchführung	Sinn
TOF Korrektur	Kurativ	VSD Patchverschluss, transpulmonale Myektomie oder Ventrikulotomie mit evtl. transanulärer Patcherweiterung	Herstellung einer normalen Anatomie und Physiologie
TGA, „arterial switch“	Kurativ	Aorta und die Pulmonalarterie werden verlagert, Transfer der Koronararterien	Herstellung einer normalen Anatomie und Physiologie
TGA, Vorhofumkehr (Senning oder Mustard)	Palliativ	Neuseptierung auf Vorhofebene mittels Verwendung nativen Vorhofmaterials oder durch Einnähen eines Perikardpatches, systemvenöses Blut wird zum LV und pulmonal venöses zum RV geleitet	Überleben ermöglichen, Zyanose beseitigen Problem: RV ist Systemventrikel mit HI im Langzeitverlauf
Fontan bei (funktionell) univentrikulärem Herzen	Palliativ	Die VCS wird als 1. Op. mit der rechten Pulmonalarterie konnektiert, als 2. Schritt wird in einer 2. Op. die VCI mit einem extrakardialen Conduit mit der rechten Pulmonalarterie verbunden	Überleben ermöglichen, Zyanose beseitigen, Verbesserung der Lungendurchblutung Problem: Fehlen des subpulmonalen Ventrikels, eine Kammer übernimmt die Funktion beider Kammern mit HI im Langzeitverlauf
BlalockTaussig Shunt	Palliativ	Anastomose der A. subclavia mit der ipsilateralen Pulmonalarterie	Verbesserung der Lungendurchblutung
Pulmonalarterienband	Palliativ	Erzeugen einer Pulmonalarterienstenose des Truncus pulmonalis	Schutz des Lungenkreislaufs vor Überflutung und Verhinderung der Entwicklung einer pulmonalen Hypertonie

*Op.* Operation, *TOF* Fallot-Tetralogie, *TGA* Transposition der großen Arterien, *HI* Herzinsuffizienz, *VCS* Vena cava superior, *VCI* Vena cava inferior, *RV* rechter Ventrikel

## Fallgruben und anatomische Besonderheiten

**Figure 4:**
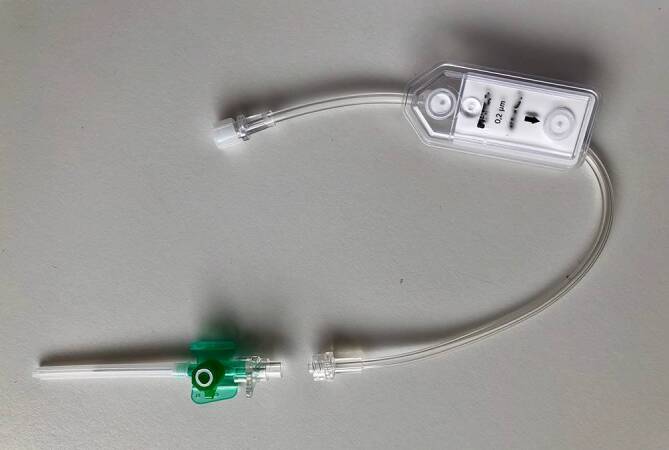

Wie sonst üblich, müssen auch bei der Beurteilung von EMAH-Patientinnen folgende Punkte berücksichtigt werden: die aktuellen Beschwerden, das vorliegende Vitium mit Voroperationen und den dadurch zu erwartenden Komplikationen (z. B. paradoxe Embolien bei Shunts) oder sonstige, nichtkardiale Vorerkrankungen. Oft finden sich deutlich veränderte Vor-EKGs (z. B. ein kompletter Rechts- oder Linkschenkelblock). Zu beachten ist beispielsweise auch, dass eine Hypovolämie von zyanotischen Patienten und von Patienten mit univentrikulärem Herzen (Fontan-Palliation) nur schlecht toleriert wird und daher rasch ausgeglichen werden muss (Dehydrierung vermeiden!). Bei Blutungen ist wichtig zu wissen, dass zyanotische Patienten ein hohes Hämoglobin für eine optimale Sauerstoffversorgung brauchen und somit ihre individuellen Transfusionsgrenzen deutlich höher liegen als die konventionellen Transfusionsgrenzen. Bei Patienten mit residualen intrakardialen oder intrapulmonalen Shunts müssen für alle venösen Infusionen Luftfilter (Abb. 4) verwendet werden, um paradoxe Luftembolien zu vermeiden. Als Luftfilter können vorhandene Filtersystem verwendet werden, die üblicherweise auf onkologischen Abteilungen (Bakterienfilter) oder auf Intensivstationen vorrätig sind. Es ist wichtig, größere Luftbläschen vom Eintritt in den venösen Kreislauf abzuhalten und dadurch paradoxe systemische Embolien zu verhindern. Zudem kann es bei Patienten mit pulmonaler Hypertonie und Shunts bei der Verabreichung von systemischen Vasodilatatoren zur Zunahme eines Rechts-Links-Shunts mit ausgeprägter Zyanose kommen.

Bei EMAH-Patienten dürfen jedoch auch nicht die sonst häufigen Ursachen für Notaufnahmeaufnahmen vernachlässigt werden, z. B. respiratorische oder Harnwegsinfektionen, Alkohol- oder Drogeneinnahme, nicht eingenommene Dauermedikation, thromboembolische Erkrankungen und mit zunehmendem Alter auch ein akutes Koronarsyndrom. Bei Fieber sollte jedoch eine Endokarditis als mögliche Ursache stets berücksichtigt werden. Insbesondere die Präsentation von Patienten mit Endokarditiden von Pulmonalklappenprothesen ist oft atypisch, und diese Endokarditis wird deshalb oft zunächst übersehen.

Anatomische Besonderheiten sind in Tab. 4 zusammengefasst. Beispielsweise hat ein Teil der Patienten mit komplexen Herzfehlern durch frühere chirurgische Eingriffe eine chronisch verschlossene linke oder rechte A. subclavia (z. B. nach klassischem Blalock-Taussig-Shunt als Palliation einer Fallot-Tetralogie oder nach Subclavian-Flap-Plastik für eine Aortenisthmusstenosekorrektur) mit Ausbildung eines Kollateralkreislaufes zur Versorgung des betroffenen Armes. Bei diesen Patienten muss die Blutdruckmessung zwingend am nichtbetroffenen Arm erfolgen. Die meisten dieser Operationen erfolgen über eine laterale Thorakotomie. Bei Patienten mit Thorakotomienarben muss deshalb der Blutdruck stets an beiden Armen gemessen werden.

**Table 4:** 

Parameter	Eingriff/Symptom	Auswirkung
Arterieller Blutdruck	Z. n. Blalock-Taussig-Shunt	Falsch-niedrig auf der betroffenen Seite, am anderen Arm messen!
	Aortenisthmusstenose	Niedrigere Werte an den unteren Extremitäten
	Morbus Eisenmenger	Arterieller Druck entspricht pulmonal arteriellem Druck
	Z. n. multiplen HK-Eingriffen femoral bzw. radial	Verschlüsse der Arterien, Kanülierung nicht möglich
Pulsoxymetrie	Zyanose	Ungenau bei S_p_O_2_ <80 %
	PDA	Messungen an den oberen Extremitäten höher als an den unteren
ZVK-Anlage	Z. n. multiplen Gefäßpunktionen, Schrittmacher	Venenverschlüsse, -stenosen oder -thrombosen
ZVD	Fontan-Zirkulation	Entspricht dem Pulmonalarteriendruck
Anlage eines transvenösen Interim-Schrittmachers	Fontan-Zirkulation, Trikuspidal- oder Pulmonalklappenatresie	Nicht möglich
Anlage eines Pulmonalarterienkatheters	Trikuspidal- oder Pulmonalklappenatresie	Nicht möglich
HZV	Intra- und extrakardiale Shunts	HZV-Messung ungenau
INR (Gerinnung)	Zyanose	Bei Hämatokrit von >60 % Abnahme in speziellen Zitrat angepassten Röhrchen erforderlich

*HK* Herzkatheter, *ZVK* zentralvenöser Katheter, *ZVD* zentralvenöser Druck, *HZV* Herzzeitvolumen, *INR* International Normalized Ratio, *PDA* persistierender Ductus arteriosus Botalli, *S*_*p*_*O*_*2*_ Sauerstoffsättigung

Bei Unklarheiten ist immer eine rasche Kontaktaufnahme am besten mit dem behandelnden EMAH-Zentrum notwendig, insbesondere bei komplexen kongenitalen Vitien, die rasch hämodynamisch stabilisiert werden müssen.

## Rhythmusstörungen als häufigste Ursache für EMAH-Notfälle

Das Auftreten von Rhythmusstörungen ist eines der Hauptprobleme in der Langzeitbetreuung von EMAH-Patienten und ist der häufigste Grund für eine stationäre Aufnahme. Arrhythmien sind einerseits die häufigste Ursache einer hämodynamischen Verschlechterung, andererseits kann auch der zugrundeliegende kardiale Defekt z. B. durch Ventrikeldilatation oder myokardialer Fibrose und durch postoperative Residuen oder Narben zum Entstehen von komplexen Arrhythmien führen. Die häufigsten Rhythmusstörungen im Rahmen von Notaufnahmen sind supraventrikuläre Tachykardien. Sehr häufig handelt es sich um typisches Vorhofflattern oder um sog. „intraatriale Reentry-Tachykardien“ (IART), die auf anderen Reentry-Kreisen im Vorhof beruhen (*atypisches* Vorhofflattern). Vorhofflimmern und andere atrioventrikuläre Reentry-Tachykardien treten seltener auf, die Häufigkeit des Vorhofflimmerns nimmt mit dem Alter zu. Ventrikuläre Tachykardien (Häufigkeit ca. 7 %) und symptomatische Bradykardien (insbesondere durch Sinusknotendysfunktion oder Schrittmacherdysfunktion) sind selten [4]. Angeborene Herzfehler mit einem deutlich erhöhten Risiko für Rhythmusstörungen sind die Transposition der großen Arterien (TGA) nach Vorhofumkehroperation (Abb. 3), die Fallot-Tetralogie (Abb. 2) und das funktionell univentrikuläre Herz nach Fontan-Operation (Abb. 1). Bei der TGA nach Vorhofumkehroperation (Abb. 3) führt die extensive Vorhofchirurgie im Langzeitverlauf häufig zu Sinusknotendysfunktion und Vorhofflattern, bei Dysfunktion des rechten Ventrikels (RV) auch zu komplexen ventrikulären Rhythmusstörungen. Bei Zustand nach operativer Reparatur der Fallot-Tetralogie (TOF; Abb. 2) stehen Vorhofflattern und ventrikuläre Tachykardien mit einem signifikanten Risiko eines plötzlichen Herztodes im Vordergrund. Beim funktionell univentrikulären Herz nach Fontan-Operation (Abb. 1) sind es vor allem supraventrikuläre Arrhythmien, insbesondere Vorhofflattern und andere Formen von IART und eine Sinusknotendysfunktion, die im Langzeitverlauf auftreten. Rhythmusstörungen finden sich auch häufig bei Erwachsenen mit unkorrigierter und reparierter Ebstein-Anomalie (in Richtung RV Apex verlagerte Trikuspidalklappe mit Verkleinerung des RV Cavum) in Form von supraventrikulären Tachykardien, häufig auf dem Boden einer akzessorischen Leitungsbahn (Wolff-Parkinson-White-Syndrom), und bei Vorhofseptumdefekten finden sich oft typisches Vorhofflattern und Vorhofflimmern [5–11].

Zu beachten ist, dass im Oberflächen-EKG bei vielen Vitien durch vorbestehende Rechts- oder Linksschenkelblockbilder supraventrikuläre von ventrikulären Tachykardien oft nicht sicher differenzierbar sind. Im Zweifel sollten alle Breitkomplextachykardien zunächst wie ventrikuläre Tachykardien behandelt werden. Äußerst hilfreich ist es in diesem Zusammenhang, ein EKG des Patienten im normalen Sinusrhythmus vorliegen zu haben. Hiermit lassen sich SVT von VT häufig rasch abgrenzen.

### Akuttherapie der Rhythmusstörungen

Grundsätzlich unterscheidet sich die Akuttherapie bei EMAH nicht von anderen Patienten mit denselben Rhythmusstörungen. Nach der akuten Behandlung sowohl von supraventrikulären als auch ventrikulären Tachykardien ist immer die Abklärung der zugrundeliegenden hämodynamischen Situation indiziert. In vielen Fällen wird eine weitere invasive Abklärung mittels Herzkatheter und ggf. eine interventionelle oder eine operative Verbesserung der hämodynamischen Situation notwendig werden. Die medikamentöse prophylaktische Langzeitbehandlung tachykarder Arrhythmien bei EMAH ist meist wenig effektiv und durch Nebenwirkungen limitiert (negativ chronotrop und dromotrop, d. h. bradykardisierend bzw. atrioventrikuläre Blockierungen fördernd; negativ inotrop). Häufig ist eine frühe elektrophysiologische Untersuchung, in der Regel verbunden mit einer Katheterablation, sinnvoll und hilfreich. Allerdings erfordern die meist komplexe Anatomie des Herzfehlers und die teilweise veränderten oder eingeschränkten Gefäßzugangswege besondere Erfahrung, sodass eine adäquate Versorgung bei komplexen Vitien nur an ausgewählten Zentren durchgeführt werden sollte. Die Erfolgsrate der Katheterablationstherapie liegt je nach Art des Vitiums und der durchgeführten Operation häufig unter der bei strukturell normalen Herzen [12].

### Tachykardien

#### Supraventrikuläre Tachykardien

Supraventrikuläre Tachykardien sind besonders häufig bei TGA nach Vorhofumkehr-Operationen (Mustard, Senning) sowie bei Patienten mit univentrikulärem Herzen nach Fontan-Operation oder Modifikationen und können rasch aufgrund des Verlustes der atrioventrikulären Synchronizität zu kardialer Dekompensation führen. Sie dürfen daher nicht als Bedarfssinustachykardien fehlinterpretiert werden. Supraventrikuläre Tachykardien bei EMAH-Patienten sind häufig durch eine relativ starre Pulsfrequenz von 100–140/min gekennzeichnet (meist Vorhofflattern mit 2:1 AV-Überleitung) und werden vom Patienten oft verspürt oder indirekt über eine Leistungseinschränkung bemerkt.

Die Therapie akuter tachykarder supraventrikulärer Tachykardien mit hämodynamischer Beeinträchtigung erfordert immer eine sofortige elektrische Kardioversion. Als medikamentöse Therapie zur akuten Behandlung hämodynamisch stabiler Patienten mit supraventrikulären Tachykardien stehen Adenosin, β‑Blocker oder Amiodaron zur Verfügung. Vor allem Klasse-Ia/Ic-Antiarrhythmika (Flecainid, Propafenon) können proarrhythmisch sein (Torsade des pointes, ventrikuläre Tachykardien) und sollten in der Akuttherapie vermieden werden. Bei Ineffektivität der Antiarrhythmika sollte stets rasch eine elektrische Kardioversion erfolgen, da das Risiko einer kardialen Dekompensation aufgrund hoher Ventrikelfrequenzen besteht. Bei hämodynamisch stabilen Patienten mit Vorhofflattern oder IART und einem implantierten Herzschrittmacher mit Vorhofsonden kann ggf. die Tachykardie mittels „atrial overdrive pacing“ über den Schrittmacher terminiert werden.

Bei anhaltenden IART oder Vorhofflimmern muss bei EMAH-Patienten von einem erhöhtem Thrombembolierisiko ausgegangen werden. Es sollte daher immer abgeklärt werden, ob bereits eine suffiziente Antikoagulation besteht bzw. diese unmittelbar eingeleitet werden. Bei hämodynamisch stabilen Patienten sollte vor elektiver elektrischer Kardioversion eine transösophageale Echokardiographie zum Ausschluss intrakardialer Thromben durchgeführt werden.

#### Ventrikuläre Tachykardien

Ventrikuläre Tachykardien treten wesentlich seltener auf und finden sich vor allem bei postoperativen TOF-Patienten oder Patienten mit TGA nach Vorhofumkehroperation. Meist ist eine rasche Kardioversion notwendig. Für die medikamentöse Akuttherapie bei hämodynamisch stabilen Patienten empfiehlt sich Amiodaron.

Zur primärprophylaktischen Implantation von implantierbaren Kardioverter-Defibrillatoren (ICD) liegen bei EMAH nur limitierte Daten vor. Eine Indikation zur Implantation bei EMAH-Patienten nach Ausschluss reversibler Ursachen ist auf jeden Fall nach überlebtem plötzlichem Herztod oder bei dokumentierten symptomatischen, anhaltenden, ventrikulären Tachykardien gegeben, die nicht durch Ablation therapiert werden können [1, 3, 13]. Die Vitien mit dem höchsten Risiko für einen plötzlichen Herztod sind TOF, TGA, hochgradige Aortenklappenstenosen und univentrikuläre Herzen [3].

### Kardioversion bei EMAH-Patienten: Fallstricke

Bei EMAH-Patienten mit Tachykardien wird eine rasche elektrische Kardioversion bei diagnostischen Unsicherheiten wegen des unsicheren Ansprechens auf die medikamentöse Standardtherapie und der Gefahr der raschen kardialen Dekompensation empfohlen. Bei komplexen Vitien ist jedoch die Auswahl des am besten geeigneten Sedativums für die Kardioversion kritisch, um eine durch Vasodilatation verursachte Verschlechterung der Hämodynamik zu verhindern. Der Notfallarzt muss auf die möglichen, zu erwartenden Probleme vorbereitet sein, um rasch gegensteuern zu können. Sedativa hemmen den Sympathikus und führen zu Vasodilatation. Dies kann zu gefährlichen Blutdruckabfällen und Abnahme der Kontraktilität des Herzens führen, bei Shuntvitien auch zu einer Zunahme der Zyanose. Die Gabe von postiv-inotrop wirksamen Katecholaminen erhöht jedoch wiederum das Risiko für Arrhythmien, sodass die Gabe eines reinen α‑Agonisten (z. B. Phenylepinephrin) nach Flüssigkeitssubstitution zur Druckstabilisierung die Therapie der Wahl ist. Prinzipiell sollte zur Sedierung nur vertraute Medikamente einsetzt werden. Beim üblicherweise verwendeten Propofol (Standarddosis für die Kardioversion: 0,5 mg/kg/KG) ist die Gefahr eines deutlichen Abfalls des peripheren Gefäßwiderstands und mittleren arteriellen Drucks zu beachten. Midazolam wird diesbezüglich meist besser toleriert. Etomidat hingegen ist zwar normalerweise hämodynamisch gut verträglich aber supprimiert die Cortisolausscheidung aus den Nebennieren. Bei kritisch Kranken insbesondere mit Infektionen kann auch die Einmalgabe zu Problemen führen.

Hinweis: Anatomische Vitium-bedingte Lagevarianten des Herzens oder Dextrokardie müssen bei der Elektrodenplatzierung am Thorax beachtet werden. Gegebenenfalls muss eine anteroposteriore Positionierung durchgeführt werden. Falls aufgrund der Anatomie des Vitiums kein Interim-Schrittmacher transvenös gelegt werden kann (z. B. Fontan-Zirkulation), erhöht dies zusätzlich das Risiko der Kardioversion im Fall einer protrahierten, symptomatischen Bradykardie nach erfolgreicher Kardioversion.

### Bradykardien

Bei symptomatischen Bradykardien (z. B. Sinusknotendysfunktion, höhergradigem AV-Block, bradykardem junktionalem Ersatzrhythmus) gelten für die Notfallversorgung die üblichen Therapierichtlinien mit Atropin, Isoproterenol und ggf. Epinephrin, transvenöse Interim-Schrittmacher oder – falls nicht möglich – perkutane Schrittmacherstimulation. Diese erfordert aber eine Analgosedierung des Patienten. Bei insuffizienter perkutaner Stimulation müssen die Elektrodenpositionen variiert werden, weil je nach zugrundeliegendem Herzfehler eine atypische Anatomie vorliegen kann. Bei Problemen ist eine rasche chirurgische Schrittmacherversorgung durch einen erfahrenen Chirurgen oder Elektrophysiologen notwendig. Es können epikardiale statt endokardialer Elektroden erforderlich werden. Wann immer möglich, sollte eine atrioventrikuläre Synchronie erreicht werden [14].

## Akute kardiale Dekompensation

Die akute kardiale Dekompensation wird bei EMAH-Patienten zunehmen, weil Patienten mit Fontan-Zirkulation oder solche mit einem morphologisch rechten als Systemventrikel fungierenden Ventrikel (z. B. TGA nach Vorhofumlagerung, kongenital korrigierte TGA) immer älter werden. Neben den allgemein gültigen Regeln der Therapie der akuten Herzinsuffizienz mit Optimierung der Vor- und Nachlast, der Herzfrequenz und Optimierung des Volumenstatus (z. B. Gabe von Diuretika bei Überwässerungszeichen, Volumensubstitution bei Hypotonie) benötigt die Therapie akut kardial dekompensierter komplexer, voroperierter oder nichtkorrigierter kongenitale Vitien die Kenntnis und die Berücksichtigung der komplexen Pathophysiologie [15]. Vitienabhängig bevorzugte Medikamente zur Aufrechterhaltung eines ausreichenden Systemdrucks im Rahmen der akuten Herzinsuffizienz sind in Tab. 5 zusammengefasst. Digitalispräparate werden nur mehr selten verwendet; sie weisen eine vergleichsweise geringe positiv inotrope Wirkung insbesondere auf den rechten Systemventrikel auf, können aber durchaus zur Herzfrequenzkontrolle bei Rhythmusstörungen in Frage kommen. Bei diuretikaresistenter Volumenüberlastung muss rasch mit einer Ultrafiltrationstherapie begonnen werden. Andererseits sollen Diuretika bei einer vorlastabhängigen Zirkulation (z. B. univentrikuläres Herz, TGA mit Vorhofumkehr-Operation) nur vorsichtig eingesetzt werden. Pleuraergüsse müssen großzügig drainiert werden. Zu bedenken ist, dass Hypoxie, Hyperkapnie, Azidose und Hypothermie sowie die Beatmung mit hohen Tidalvolumina oder hohen positiv endexpiratorischen Druckwerten den pulmonalen Gefäßwiderstand weiter erhöhen und daher möglichst zu vermeiden sind. Eine assistierte Spontanatmung sollte möglichst rasch angestrebt werden. Falls die akute Therapie nicht rasch den gewünschten Erfolg zeigt, sollen die Patienten in ein EMAH-Zentrum verlegt werden. Bei Patienten, die prinzipiell für eine interventionelle oder operative Behandlung oder Herz- bzw. Herz-Lungen-Transplantation in Frage kommen, müssen beispielsweise mechanische Kreislaufunterstützungsverfahren (z. B. extrakorporale Membranoxygenierung [ECMO] oder „ventricular assist devices“) rechtzeitig initiiert werden, bevor der Patient ein möglicherweise irreversibles Multiorganversagen entwickelt.

**Table 5:** 

Vitium	HF	Kontraktilität	Vorlast	Nachlast	Therapieziele, Probleme	Bevorzugte Medikamente zur Aufrechterhaltung eines ausreichenden systemischen arteriellen RR
Shuntvitium (z. B. ASD, VSD)	*n*/↑	*n*/↑	↑	↓	CAVEAT: Zunahme des Links-Rechts-Shunts bei Druckzunahme in den linken Herzhöhlen	Dobutamin PDE-Hemmer (Levosimendan)
Zyanotische Shuntvitien	*n*/↑	↑	*n*/↑	↓ (pulmonal) *n*/↑ (systemisch)	Senkung des Pulmonalgefäßwiderstands, systemische Vasodilatation vermeiden (Gefahr der Zunahme des Shunts)	Pulmonale Vasodilatatoren, Dobutamin (evtl. mit α‑Agonisten), Epinephrin
Fallot-Tetralogie, Z. n. Op.	↑	*n*/↑	↑	↓	PI führt zu chron. RV-Insuffizienz Dynamische subvalvuläre RV-Obstruktion ausschließen	Dobutamin, PDE-Hemmer (Levosimendan)
TGA mit Z. n. Vorhofumkehrkorrektur Op. (Senning, Mustard)	↑	*n*/↑	↑	↓	RV = Systemventrikel, chron. Insuffizienz, sekundäre TI CAVEAT: Sinusknotendysfunktion, Shunts, Baffle-Obstruktionen, subpulmonale Obstruktion	Dobutamin, PDE-Hemmer (Levosimendan)
Fontan-Zirkulation (funktionell univentrikuläres Herz)	*n*/↑	*n*/↑	↑	*n*	Die Senkung des pulmonalen Gefäßwiderstands ist essenziell	Pulmonale Vasodilatatoren
Dynamische intraventrikuläre Obstruktion	↓	↓	↑	↑	Positiv inotrope Medikamente, Tachykardie und systemische Vasodilatation vermeiden, Volumengabe zur Vorlastoptimierung, normofrequenten SR erhalten	Betablocker, Phenylepinephrin Norepinephrin

*ASD* Vorhofseptumdefekt, *VSD* Ventrikelseptumdefekt, *PDE* Phosphodiesterase, *n* normal, *Op.* Operation, *PI* Pulmonalklappeninsuffizienz, *RV* rechter Ventrikel, *TGA* Transposition der großen Arterien, *TI* Trikuspidalklappeninsuffizienz, *SR* Sinusrhythmus

Einige vitiumtypische Besonderheiten sind beispielsweise der pulmonale Gefäßwiderstand (z. B. bei zyanotischen Vitien, Fontan-Zirkulation oder pulmonaler Hypertonie), der nicht gesteigert werden darf (Caveat: Noradrenalingabe, Beatmung mit hohen Beatmungsdrücken oder positiv endexpiratorischem Druck), sondern gesenkt werden muss (z. B. mit Epoprostenol oder Stickstoffmonoxid). Bei pulmonaler Hypertonie kommt Argipressin (Vasopressin) bei längerem Bedarf eines systemischen Vasokonstriktors zum Einsatz, weil die Wirkung im Systemkreislauf deutlich stärker als im Pulmonalkreislauf ist [16]. Bei Fontan-Kreislauf ist zu beachten, dass bei diesen Patienten der zentralvenöse Druck dem pulmonalarteriellen Druck entspricht. Die Vorlastoptimierung durch Volumengabe ist wichtig, um den Systemdruck zu stabilisieren, da die Kontraktilität des Systemventrikels üblicherweise nicht die Ursache der Hypotonie ist. Eine Lungenembolie als mögliche Ursache der akuten Dekompensation darf nicht übersehen werden.

Shuntvitien (z. B. Vorhofseptum- und Ventrikelseptumdefekte) können über eine jahrzehntelange chronische Volumenbelastung zu einer chronischen Herzinsuffizienz mit einer Zunahme des pulmonalen Gefäßwiderstands führen. Bei zyanotischen Vitien sollte das Gleichgewicht zwischen systemischem und pulmonalem Widerstand aufrechterhalten werden. Falls der periphere Gefäßwiderstand sinkt (z. B. bei Sepsis, Hitze, Gabe von vasodilatierenden Sedativa) und der pulmonale Widerstand dabei gleichbleibt oder sogar steigt, verstärkt sich die (Misch‑)Zyanose.

Das häufigste mittelkomplexe Vitium in der klinischen Praxis sind Patienten nach operativer Korrektur einer TOF. Eine residual hochgradig insuffiziente Pulmonalklappe kann über eine RV-Volumenbelastung und Dysfunktion zu einer chronischen Rechtsherzinsuffizienz führen. Eine akute Dekompensation wird nach den üblichen Richtlinien therapiert (Diuretika, positiv inotrope Therapie, Herzfrequenzkontrolle), bevor definitive interventionelle oder chirurgische Eingriffe in Frage kommen.

Bei TGA mit Z. n. Vorhofumkehr-Operation oder bei kongenital korrigierter TGA fungiert der morphologisch rechte Ventrikel als Systemventrikel. In einem Teil der Fälle entwickelt sich über Jahrzehnte eine chronische Herzinsuffizienz mit sekundärer Trikuspidalklappeninsuffizienz. Eine akute kardiale Dekompensation wird nach den üblichen Richtlinien behandelt (Tab. 5). Dabei ist zu beachten, dass die Patienten von einer Dauertherapie mit ACE-Hemmern möglicherweise nicht oder weniger profitieren als Patienten mit nichtangeborenen Herzfehlern. Stenosen in den Tunnels („Baffles“) müssen als Ursache der Herzinsuffizienz ausgeschlossen werden.

Bei EMAH-Patienten dürfen neben den vitientypischen Aspekten jedoch im Rahmen der Abklärung der akuten Herzinsuffizienz nicht die sonst üblichen Ursachen einer kardialen Dekompensation vernachlässigt werden, insbesondere mit zunehmendem Alter der Patienten. Es ist immer auch beispielsweise an eine Lungenembolie, Myokardischämie (besonders bei TGA und Zustand nach Arterial-switch-Operation, Abb. 3), Sepsis, akutes Lungenversagen durch eine Pneumonie oder an eine Perikardtamponade zu denken.

## Weitere, jedoch wesentlich seltenere EMAH-Notfallsituationen

### Hämoptysen

Hämoptysen sind seltene, jedoch für die Patienten subjektiv sehr bedrohliche Notfallsituationen. Das Ausmaß der Hämoptysen wird nicht selten überschätzt, sie werden nach der Menge des Blutverlustes in leicht (<100 ml), mittel (100–300 ml) und massiv (>300 ml) eingeteilt. Der Blutverlust ist selten bedrohlich und die Hämodynamik nur selten beeinträchtigt, aber bei zyanotischen Patienten mit pulmonaler Hypertonie sind Hämoptysen eine relevante Todesursache. Die fachgerechte Notfallversorgung und anschließende Kontaktaufnahme mit einem EMAH-Zentrum sind daher besonders wichtig. Verursacht werden Hämoptysen bei zyanotischen Vitien durch Pulmonalembolien meist aus In-situ-Thrombusformationen in den Pulmonalarterien (Pulmonalembolien aus venösen Thromben sind vergleichsweise selten) oder Blutungen aus pulmonalen arteriovenösen Gefäßmissbildungen oder Kollateralgefäßen. Des Weiteren können auch pulmonale Infektionen zu Hämoptysen führen. Nach den üblichen Erstmaßnahmen mit ggf. notwendiger Intubation des Patienten muss rasch eine CT-Angiographie der Pulmonalstrombahn durchgeführt werden, um die Blutungsquelle zu lokalisieren und falls möglich interventionell im Anschluss durch gezielte Embolisation (z. B. Coiling) des zuführenden Gefäßes zu stoppen. Es ist zu beachten, dass Notfallbronchoskopien zur Blutungsstillung bei EMAH-Patienten mit Hämoptysen die Blutung sogar verschlechtern können. Inhalationen mit z. B. Tranexamsäure können versucht werden (Caveat: Zunahme des Hustenreizes mit Gefahr der Zunahme der Blutung). Bei der Erstversorgung von Eisenmenger-Patienten mit Hämoptysen ist die Senkung eines erhöhten systemischen Blutdrucks durch Sedativa und β‑Blocker wichtig (Caveat: Systemische Vasodilatatoren können zur Zunahme des Rechts-Links-Shunts führen), weil dadurch auch der Druck im blutenden Pulmonalgefäß gesenkt werden kann. Zur Therapieüberwachung eignet sich gut der arterielle Blutdruck, der auch in etwa dem pulmonal arteriellen Druck bei Eisenmenger-Patienten entspricht. Alle venösen Zugänge müssen mit Luftfiltern versehen werden, um paradoxe Embolien zu verhindern.

Hämoptysen und Hämatemesis bei Patienten nach operativer Reparatur einer Aortenisthmusstenose können durch Aneurysmabildung im Operationsgebiet mit der Ausbildung von aortobronchialen bzw. aortoösophagealen Fisteln verursacht werden. Vor Endoskopien müssen diese Ursachen durch eine CT-Untersuchung ausgeschlossen werden.

### Akut neu aufgetretene Kopfschmerzen

Diese Patienten brauchen eine kurzfristige Abklärung mittels bildgebender Verfahren, um einen Hirnabszess (besonders bei gleichzeitigem Fieber) bei bestehendem Rechts-Links-Shunt, einen ischämischen Insult durch paradoxe Embolie oder Erythrozytose oder eine zerebrale Blutung bei Gerinnungsstörung (z. B. bei oraler Antikoagulation oder bei Eisenmenger-Syndrom) auszuschließen.

Zusätzlich leiden Patienten mit zyanotischen Vitien oft an chronischen Kopfschmerzen, die durch eine zerebrale Mikrozirkulationsstörung bedingt sind und nur schlecht auf die üblichen Schmerzmedikamente ansprechen. Es sollte – soweit noch möglich – die Therapie der pulmonalen Hypertonie zur Verbesserung der Oxygenation optimiert werden und auf eine ausreichende Flüssigkeitszufuhr geachtet werden (angestrebter Hämatokrit <65 %). Phlebotomien sind nur sehr selten indiziert, z. B. bei akuter neurologischer Symptomatik und einem Hämatokritwert >65 % mit normalem Eisenstatus unter entsprechender Flüssigkeits- und Eisensubstitution [1, 3]. Ansonsten können sie den Patienten eher schaden (durch Eisenmangel und Verstärkung der Hyperviskosität).

### Endokarditis

Das Endokarditisrisiko der EMAH-Patienten ist wesentlich höher als das der Allgemeinbevölkerung. Das höchste Risiko wiederum weisen Patienten mit Klappen- und sonstigen Prothesen, Patienten mit Endokarditisvorgeschichte, Patienten mit Klappen- (z. B. bikuspide Aortenklappe) oder zyanotischen Vitien und Patienten mit Residualdefekten (z. B. Ventrikelseptumdefekt), palliativen Shunts (z. B. extrakardiale Endokarditis bei Z. n. Blalock-Taussig-Shunt) oder Conduits auf. Primär wird zum Nachweis und zur Ermittlung des Ausmaßes einer kardialen Beteiligung eine transösophageale Echokardiographie durchgeführt. Bei klinischer Verschlechterung von Vitienpatienten oder kardialer Dekompensation muss auch an eine Endokarditis als zugrundeliegende Ursache gedacht werden. Zunehmend häufiger beobachtet werden Endokarditiden bei Patienten nach prothetischem Pulmonalklappenersatz. Diese können sich atypisch präsentieren, z. B. primär mit septischen Lungenembolien, weshalb die Diagnosestellung verzögert sein kann. Oft kommt es bei diesen Patienten zudem zu einer rasch progredienten Stenose der Pulmonalklappenprothese, die rasch zu einer hämodynamischen Instabilität bis zum kardiogenen Schock führen kann.

### Aortendissektion

Bei Patienten mit Bindegewebserkrankungen (z. B. Marfan-Syndrom) und Voroperationen oder Interventionen im Bereich der Aorta soll die Aortendissektion immer in die differenzialdiagnostischen Überlegungen zur Abklärung von Thoraxschmerzen miteinbezogen werden. Eine rasche über die Echokardiographie hinausgehende Bildgebung ist dazu notwendig (z. B. Computertomographie-Angiographie).

### „Plastic bronchitis“

Bei Fontan-Patienten kommt es gelegentlich zum Austritt von Eiweiß in das Bronchialsystem, das als weißliche, kleine, wurmartige Gebilde („casts“, ähnlich Plastikröhrchen) ausgehustet wird. Das Verlegen größerer Bronchien kann akute Hypoxämie hervorrufen. Die Akutversorgung soll mit dem betreuenden EMAH-Zentrum abgesprochen werden (z. B. lokale Lyse der „casts“ durch Inhalation eines Thrombolytikums, bronchoskopische Extraktion).

## Fazit für die Praxis

- Die Zahl der Aufnahmen von EMAH-Patienten an Notfallaufnahmen außerhalb von spezialisierten Zentren ist noch niedrig, wird aber kontinuierlich zunehmen.
- Ihr Management erfordert spezielles Wissen.
- Das Wesentliche wurde in dieser Übersicht zusammengefasst.
- Eine gute rasche Erstversorgung hilft, weitere vermeidbare Komplikationen zu verhindern.
- Es ist wichtig, einen EMAH-Spezialisten (idealerweise vom behandelnden Zentrum) sofort zu konsultieren, wenn sich der Notfall nicht rasch beherrschen lässt bzw. auch im Anschluss nach erfolgreicher Stabilisierung des Patienten.
